# Supplementary material for: Near 100% efficient homology-dependent genome engineering in the human fungal pathogen Cryptococcus neoformans
Source: G3 (Bethesda). 2025 Jun 3;15(8):jkaf118. doi: 10.1093/g3journal/jkaf118 (PMC12341884; doi:10.1093/g3journal/jkaf118)
Supplement: jkaf118_Supplementary_Data [file jkaf118_supplementary_data.zip › Supplemental_Figures_Legends_G3-2025-405906.docx]

Figure S1. Creating the *yku80*-blaster allele and restoring *YKU80*

The 313-bp blaster region spans L23 in exon 1 through S92 in exon 3 and includes introns 1 and 2 (genomic coordinates: Chr 2, 405251-405563); cyan exon boxes denote the blaster region; triangle flags indicate direct repeats. The introduced blaster sequence includes two silent mutations (indicated by red bases), which swap codons of S91 and S92; these base changes disrupt the PAM and guide target highlighted in the box (see also Fig. S2).

Figure S2. Sequences of *YKU80* blaster region

Alignments of *YKU80* reference sequence and Sanger sequencing data from “Restored A” and “Restored B” strains after removal of *amdS* from the *yku80*-blaster allele. The 313-bp blaster sequence is indicated by the cyan boxes. The guide target and PAM are outlined with red boxes, and trace data from Sanger sequencing are included below them.
