# Supplementary figures and images for: Near 100% efficient homology-dependent genome engineering in the human fungal pathogen Cryptococcus neoformans
Source: G3 (Bethesda). 2025 Jun 3;15(8):jkaf118. doi: 10.1093/g3journal/jkaf118 (PMC12341884; doi:10.1093/g3journal/jkaf118)

Figure S1

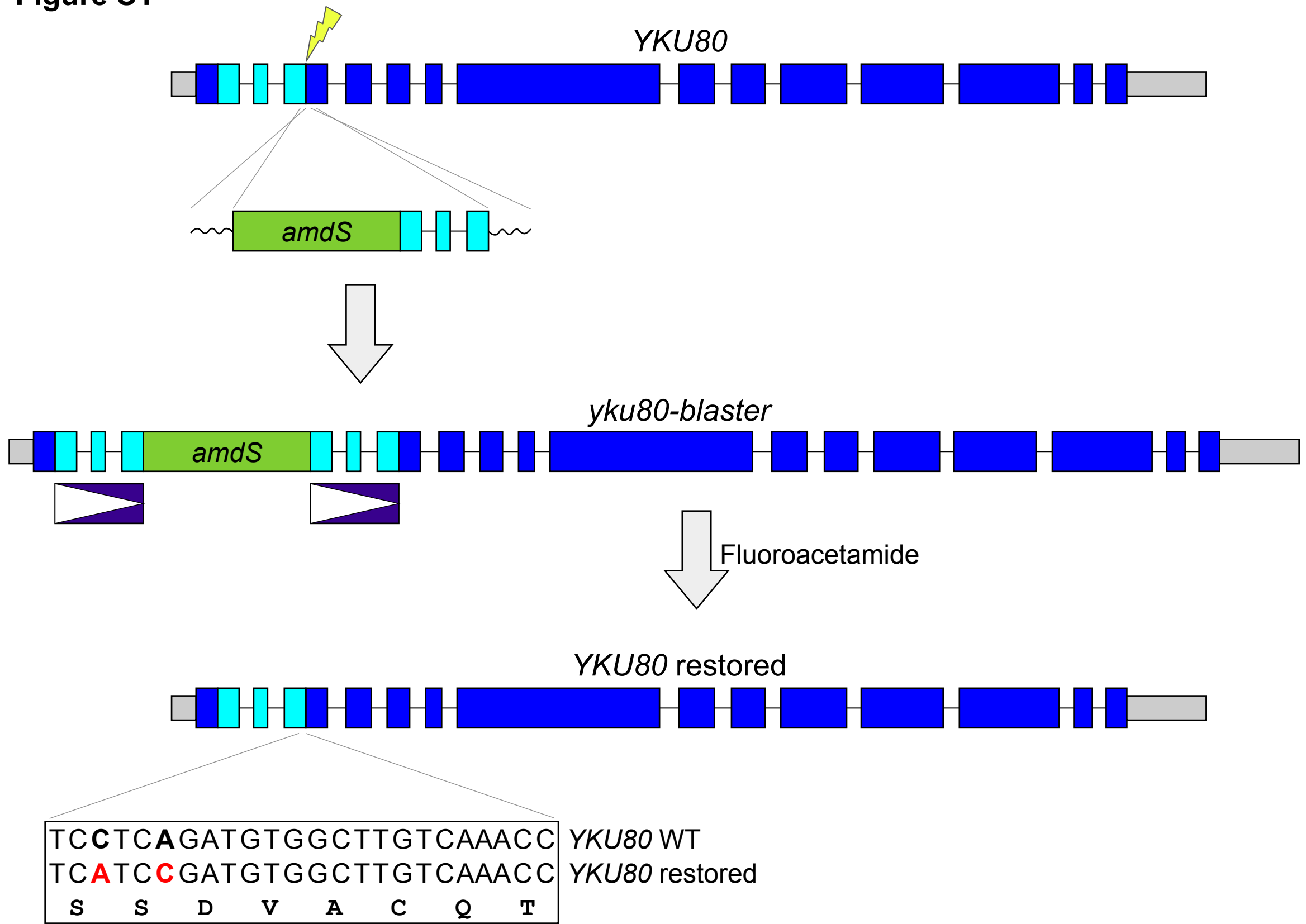

Supplement: jkaf118_Supplementary_Data [file jkaf118_supplementary_data.zip › Figure_S1_G3-2025-405906.pdf]

## Figure S2

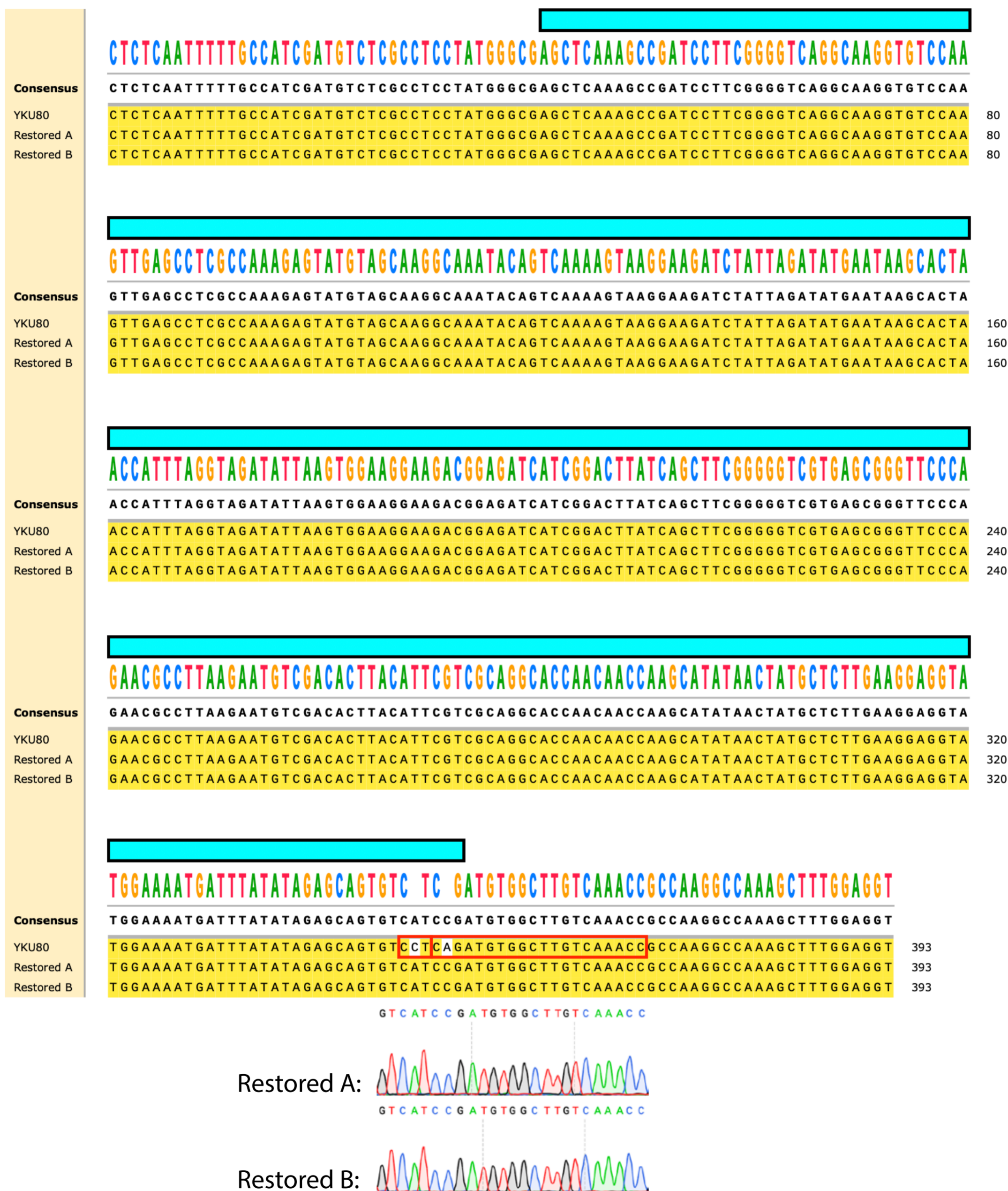

Supplement: jkaf118_Supplementary_Data [file jkaf118_supplementary_data.zip › Figure_S2_G3-2025-405906.pdf]
